# Identification and functional characterization of the sugarcane (*Saccharum* spp.) AMT2-type ammonium transporter ScAMT3;3 revealed a presumed role in shoot ammonium remobilization

**DOI:** 10.3389/fpls.2023.1299025

**Published:** 2023-11-30

**Authors:** Rodolfo A. Maniero, Alessandra Koltun, Marielle Vitti, Bruna G. Factor, Nathalia de Setta, Amanda S. Câmara, Joni E. Lima, Antonio Figueira

**Affiliations:** ^1^ Centro de Energia Nuclear na Agricultura, Universidade de São Paulo, Piracicaba, SP, Brazil; ^2^ Centro de Ciências Naturais e Humanas, Universidade Federal do ABC, São Bernardo do Campo, SP, Brazil; ^3^ Genebank Department, Leibniz Institute of Plant Genetics and Crop Plant Research (IPK), Seeland, Germany; ^4^ Instituto de Ciências Biológicas, Universidade Federal de Minas Gerais, Belo Horizonte, MG, Brazil

**Keywords:** *Arabidopsis thaliana,* heterologous expression, low-affinity transport system, nitrogen, nitrogen use efficiency, *Saccharomyces cerevisiae*

## Abstract

Sugarcane (*Saccharum* spp.) is an important crop for sugar and bioethanol production worldwide. To maintain and increase sugarcane yields in marginal areas, the use of nitrogen (N) fertilizers is essential, but N overuse may result in the leaching of reactive N to the natural environment. Despite the importance of N in sugarcane production, little is known about the molecular mechanisms involved in N homeostasis in this crop, particularly regarding ammonium (NH_4_^+^), the sugarcane’s preferred source of N. Here, using a sugarcane bacterial artificial chromosome (BAC) library and a series of *in silico* analyses, we identified an *AMMONIUM TRANSPORTER* (*AMT*) from the AMT2 subfamily, sugarcane *AMMONIUM TRANSPORTER 3;3* (*ScAMT3;3*), which is constitutively and highly expressed in young and mature leaves. To characterize its biochemical function, we ectopically expressed *ScAMT3;3* in heterologous systems (*Saccharomyces cerevisiae* and *Arabidopsis thaliana*). The complementation of triple *mep* mutant yeast demonstrated that ScAMT3;3 is functional for NH_3_/H^+^ cotransport at high availability of NH_4_^+^ and under physiological pH conditions. The ectopic expression of *ScAMT3;3* in the *Arabidopsis* quadruple *AMT* knockout mutant restored the transport capacity of ^15^N–NH_4_^+^ in roots and plant growth under specific N availability conditions, confirming the role of ScAMT3;3 in NH_4_^+^ transport *in planta*. Our results indicate that ScAMT3;3 belongs to the low-affinity transport system (*K_m_* 270.9 µM; *V_max_* 209.3 µmol g^−1^ root DW h^−1^). We were able to infer that ScAMT3;3 plays a presumed role in NH_4_^+^ source–sink remobilization in the shoots via phloem loading. These findings help to shed light on the functionality of a novel AMT2-type protein and provide bases for future research focusing on the improvement of sugarcane yield and N use efficiency.

## 1 Introduction

Sugarcane (*Saccharum* spp.) is a valuable crop that plays a significant role in global sugar and bioethanol production. Currently, Brazil stands as the world’s largest producer of sugarcane, also holding the first and second positions among sugar and bioethanol producers, respectively. With the ever-increasing demand for food and energy production worldwide, it is expected that sugarcane cultivation will continue to expand to non-forested areas (de Andrade Junior et al., 2021; Zhong et al., 2021). To maintain and increase sugarcane yields, particularly in marginal areas, the use of nitrogen (N) fertilizer is essential. However, this practice has been associated with significant leaching of reactive N to the natural environment (Black et al., 1985; Galloway et al., 2014). Previous studies have shown that more than 70% of the total N applied to sugarcane fields is lost to the atmosphere in the form of ammonia (NH_3_) and N oxide gases (NO, N_2_O, and NO_2_), immobilized in the soil, or leached into waterways, causing serious environmental problems (Otto et al., 2016).

Although sugarcane roots exhibit a physiological preference for ammonium (NH_4_^+^) uptake, as opposed to nitrate under either N-sufficient or N-limited conditions (Robinson et al., 2011; Lima et al., 2022), the reasons behind the low N uptake by this crop remain elusive (Thorburn et al., 2017; de Castro et al., 2018; Lima et al., 2022). Therefore, it is important to investigate the mechanisms involved in the processes of sugarcane N homeostasis, including uptake, transport, allocation, and remobilization, to develop new cultivars with improved N use efficiency (NUE).

Ammonium transporter/methylammonium permease/Rhesus factor family (AMT/MEP/Rh family) proteins play a crucial role in regulating the homeostasis of NH_4_^+^ in many organisms (Ludewig et al., 2007). In plants, AMT transporters have been extensively studied mainly in model species, particularly *Arabidopsis thaliana*, and various crops of economic importance, such as tomato, rice, maize, sorghum, and wheat, since their first identification in yeast (*Saccharomyces cerevisiae*) (Marini et al., 1994; Gazzarrini et al., 1999; von Wirén et al., 2000; Suenaga et al., 2003; D’Apuzzo et al., 2004; Couturier et al., 2007; Gu et al., 2013; Koegel et al., 2013). AMTs can be categorized into two subfamilies: AMT1 and AMT2. AMT1s are more closely related to cyanobacterial transporters, while AMT2s are more closely related to Mep1, 2, and 3 from *S. cerevisiae* and to AmtB from *Escherichia coli* (Ludewig et al., 2001; McDonald et al., 2012; Von Wittgenstein et al., 2014); the number of AMT homologs varies largely among plant species.

Several studies have suggested that AMT family members have distinct roles in plant physiology, depending on their expression patterns, affinity to NH_4_^+^, cotransport of NH_3_/H^+^, and temporal regulation (Gazzarrini et al., 1999; Loqué et al., 2006; Ludewig et al., 2007; Yuan et al., 2007; Lima et al., 2010; Neuhäuser and Ludewig, 2014). In *Arabidopsis*, six AMTs have been identified, four of which, AtAMT1;1, AtAMT1;2, AtAMT1;3, and AtAMT1;5, account for up to 95% of the total NH_4_^+^ uptake in roots (Loqué et al., 2006; Yuan et al., 2007). AtAMT1;4 is responsible for NH_4_^+^ uptake in pollen grains (Yuan et al., 2009), whereas AtAMT2;1 mediates long-distance NH_4_^+^ translocation from roots to shoots (Giehl et al., 2017).

Six *AMT* genes have been identified in the *Saccharum spontaneum* ‘AP85–441’ genome and categorized into three groups, namely, *AMT2*, *AMT3*, and *AMT4* (Wu et al., 2021). Expression analysis has indicated that *AMT* genes show dynamic expression in roots and different segments of leaves in *S. spontaneum* ‘SES-208’ and *Saccharum officinarum* ‘LA-Purple’ (Wu et al., 2021). Our group identified and functionally characterized the ScAMT2;1 transporter from sugarcane, providing evidence that this transporter plays a significant role in NH_4_^+^ root-to-shoot translocation, potentially via xylem loading (Koltun et al., 2022). This finding sheds new light on the regulation of NH_4_^+^ homeostasis in sugarcane by AMT2-type proteins. Although the AMT3 and AMT4 groups of AMT2-type transporters in plants have been linked to mycorrhizal NH_4_^+^ transfer in a few studies (Koegel et al., 2017; You et al., 2020; Hui et al., 2022), information on the physiological functions of AMT3 and AMT4 proteins in sugarcane remains limited.

Commercial sugarcane cultivars derive from interspecific crosses between the domesticated high-sucrose noble *S. officinarum* (2*n* = 8*x* = 80) and the wild-type species *S. spontaneum* (2*n* = 8*x* = 64), followed by backcrosses to the noble sugarcane, resulting in polyploid/aneuploid hybrid cultivars (2*n* = 8*x* = 80–120) (Garsmeur et al., 2011). The genome size is estimated to be approximately 10 Gb (Hoarau et al., 2001; Le Cunff et al., 2008), with unequal contributions from the *S. officinarum* (80%–90%) and *S. spontaneum* (10%–20%) parental genomes. For this reason, sugarcane has always posed a challenge regarding genetics and genome assembly. However, with the release of the genome sequence of one *S. spontaneum* double haploid genotype (Zhang et al., 2018), we were able to validate the sugarcane *AMMONIUM TRANSPORTER 3;3* (*ScAMT3;3*) identified in a bacterial artificial chromosome (BAC) library (Tomkins et al., 1999). Subsequently, by analyzing *ScAMT3;3* expressions in various tissues and under various N conditions, we concluded that *ScAMT3;3* transcripts accumulated at high levels in aboveground tissues, particularly leaves, but they were not regulated by plant N status. Furthermore, we studied the biochemical properties and physiological function of *ScAMT3;3* using the complementation of two heterologous systems (*S. cerevisiae* and *A. thaliana*) carrying AMT knockout mutations, both defective for high-affinity transport of NH_4_^+^. Our findings suggest that ScAMT3;3 functions as a low-affinity transporter that plays a potential role in regulating the reallocation of NH_4_^+^ in leaves. Our work provides information that can further support breeding efforts to improve sugarcane NUE.

## 2 Materials and methods

### 2.1 Identification and *in silico* analysis of *ScAMT3;3*

*ScAMT3;3* sequences were retrieved from an ‘R570’ cultivar BAC genomic library (Tomkins et al., 1999) by performing qPCR using primers (GGCAGCATCGTGAAGAAGAA, CCACACCACCCAGCAGA, amplicon of 141 bp) designed based on homologous sequences from *Arabidopsis* (TAIR) and sorghum (Koegel et al., 2013). BAC clones were selected, sequenced, assembled, and annotated as previously reported (de Setta et al., 2014). For phylogenetic analysis, AMT protein sequences of *Arabidopsis*; AMT2-type sequences of sorghum, rice, and maize; the AMT3;3 sequence of *S. spontaneum* [Sspon_04G0010470_2B; Wu et al., 2021]; and the AMT sequences found in the BAC clones ‘023_O13’ and ‘178_C24’ (NCBI accession id OR413321 and OR413322, respectively) were aligned using MAFFT 7 (Katoh and Standley, 2013). The alignment was imported into MEGA X (Kumar et al., 2018), followed by phylogenetic tree construction using the neighbor-joining (NJ) method with default parameter settings and bootstrap analysis with 1,000 replicates. The ScAMT3;3 conserved domains were identified using the Conserved Domain Database (CDD; https://www.ncbi.nlm.nih.gov/Structure/cdd/cdd.shtml). The sequence logo for the AMT signature motif was created using the WebLogo v2.8.2 server (https://weblogo.berkeley.edu/logo.cgi), based on the Prosite entry access PS01219. The TMHMM Server v.2.0 (http://www.cbs.dtu.dk/services/TMHMM/) was used to predict the transmembrane domains of ScAMT3;3. The protein structure prediction of the ScAMT3;3 trimer was performed using a locally installed AlphaFold2 with its full database and multimer option (Jumper et al., 2021). Confidence scores (predicted local distance difference test (pLDDT)) and predicted aligned errors (PAEs) were retrieved using a Python script based on ColabFold code (https://github.com/amandascamara/AlphaFold_analysis). Protein structure images were generated using PyMOL v2.5 (DeLano, 2002).

### 2.2 Expression analysis of *ScAMT3;3* in sugarcane

The expression profile of *ScAMT3;3* was analyzed in various sugarcane tissues and response to N status and source. Sugarcane plants (cv. SP80-3280) were cultivated for 2 months in a nutrient solution (Loqué et al., 2006). Plants were then transferred and maintained in a solution supplied with 1 mM NH_4_NO_3_ (+N), 2 mM NH_4_Cl (NH_4_^+^), or no N (−N) for 14 d. Roots, culms, and leaf tissues, including young leaves (leaf +1) and mature leaves (leaf +3), were collected for total RNA extraction (Leal et al., 2007). cDNA synthesis was performed using SuperScript III Reverse Transcriptase (Invitrogen, Waltham, MA, USA) according to the manufacturer’s instructions. Semiquantitative RT–PCR and RT–qPCR were conducted using KAPA SYBR FAST qPCR Master Mix (2X) (Kapa Biosystems, Wilmington, MA, USA) in a RotorGene 6000 thermocycler (Corbett Research, Mortlake, NSW, Australia). Specific primers for *ScAMT3;3* were designed based on the consensus coding sequence of both BAC clones. Specific primers were used to quantify *ScAMT3;3* transcript accumulation (CCGGACTCCAGAGGTGCATT, AACGCCGCTATGTCTGCTCT, amplicon of 267 bp) using sugarcane *Ubiquitin2* (*ScUBQ2*; CTTCTTCTGTCCCTCCGATG, TCCAACCAAACTGCTGCTC, amplicon of 149 bp) as a gene reference. Amplification was performed at 50°C for 10 min and 95°C for 2 min, followed by 40 cycles of 95°C for 20 s, 60°C for 25 s, and 72°C for 25 s. The 2^−ΔΔCQ^ method was used to analyze the RT–qPCR data (Livak and Schmittgen, 2001). For semiquantitative RT–PCR, reactions were run for 24 cycles for both *ScAMT3;3*, and *ScUBQ2* genes. Data are expressed as the mean ± standard deviation (SD) (*n* = 4 biological replicates).

### 2.3 Cloning and expression vector construction

The promoter sequence of *ScAMT3;3* from the BAC clone 013_O23 and the coding sequence of *ScAMT3;3* from cDNA of sugarcane cv. SP80-3280 were amplified using a HiFi KAPA HiFi PCR Kit (Kapa Biosystems) with specific primers *proScAMT3;3* (AAAGGCATCTAAACAAGACCTCGA, GGCTGGGGCACTGGATCG, amplicon of 1,877 bp) for the promoter region and *ScAMT3;3* (ATGGCAGCAGGTGCGGTAC, TCAAACATTCTGTGTGACTCCTACAGC, amplicon of 1,452 bp) for the coding region. The amplified fragments were cloned into the pCR8/GW/TOPO entry vector (Invitrogen) and sequenced in an ABI 3500 Genetic Analyzer (Applied Biosystems, Foster City, CA, USA). A primer sequence (AGTGCCCCAGCCATGGCAGCAGGT) was designed for the concatenation of promoter and coding sequences. The resulting fragment (3,329 bp) was cloned into the pCR8/GW/TOPO (Invitrogen) entry vector and sequenced.

For yeast complementation, the coding sequences of *AtAMT1;1* and *ScAMT3;3* were amplified by PCR using the Kapa HiFi PCR Kit (Kapa Biosystems) with specific primers containing restriction sites (*AtAMT1;1*, GAATTCATGTCTTGCTCGGCCAC, CTCGAGTCAAACCGGAGTAGGTG, amplicon of 1,518 bp; *ScAMT3;3*, ACTAGTATGGCAGCAGGTGCGGTA, CTCGAGTCAAACATTCTGTGTGACTC, amplicon of 1,464 bp). The fragments were cloned into pGEM-T Easy (Promega, Madison, WI, USA) and analyzed by sequencing. Positive clones were digested (*Eco*RI and *Xho*I for *AtAMT1;1*; *Spe*I and *Xho*I for *ScAMT3;3*), and fragments were ligated into the pDR196 expression vector (Rentsch et al., 1995), generating *mep::AtAMT1;1* and *mep::ScAMT3;3* expression vectors.

For heterologous expression in *Arabidopsis*, the promoter, the coding sequence, or both sequences cloned into the pCR8/GW/TOPO entry vector were recombined into pMDC expression vectors (Curtis and Grossniklaus, 2003) using Gateway LR Clonase II enzyme mix (Invitrogen), generating the expression vectors *proScAMT3;3::*GUS (pMDC164), *35S::ScAMT3;3* (pMDC32), and *proScAMT3;3::ScAMT3;3* (pMDC99), respectively.

### 2.4 Yeast transformation and complementation analysis

The triple mutant for NH_4_^+^ transport in *S. cerevisiae* strain 31019b (*MATa ura3 mep1Δ mep2Δ::LEU2 mep3Δ::KanMX2*) (Marini et al., 1997), generously provided by Prof. Dr. Nicolaus von Wirén (IPK, Gatersleben, Germany), was genetically transformed with *mep::AtAMT1;1* or *mep::ScAMT3;3* expression vectors using the lithium acetate/single-stranded carrier DNA/PEG method (Gietz and Schiestl, 2007). To evaluate complementation of the triple *mep* mutant yeast, cellular suspensions expressing *AtAMT1;1* or *ScAMT3;3* were serially diluted from 1 to 10^−3^ and plated onto yeast nitrogen medium (YNB) without amino acids or supplemented with ammonium sulfate (Sigma, St. Louis, MO, USA) or with distinct N sources and concentrations (Dubois and Grenson, 1979). The N sources used were 0.2–20 mM NH_4_Cl, 100 mM methylammonium (MeA) with 0.1% arginine, or 1 mM arginine (Arg) as a positive control for growth. To test the growth response at increasing pH levels, 20 mM MES-Tris buffer solution at pH 5, 6, or 7.5 was used. The yeast cell growth was visually evaluated, and it was assessed in relation to the uptake of NH_4_^+^. Relative yeast growth for the 10^−1^ dilution was assessed using a quantitative imaging-based protocol (Petropavlovskiy et al., 2020).

### 2.5 Plant transformation and complementation analysis

The *Arabidopsis* quadruple mutant (*qko*) for NH_4_^+^ transport (Yuan et al., 2007), kindly provided by Prof. Dr. Nicolaus von Wirén (IPK), and *Arabidopsis* Col-0 plants (T_0_) were transformed using a floral dip method (Clough and Bent, 1998) with the vectors *proScAMT3;3::GUS* (pMDC164), *35S::ScAMT3;3* (pMDC32), and *proScAMT3;3::ScAMT3;3* (pMDC99). Seeds were selected on half-strength Murashige and Skoog (½ MS) medium with 1% (w/v) sucrose and agar and 25 mg L^−1^ hygromycin A. Approximately six T_1_ transgenic seedlings were grown to maturity in soil, and T_2_ seeds were harvested. T_2_ plants were selected on ½ strength MS medium containing 25 mg L^−1^ hygromycin A. T_2_ seedlings were grown to maturity, and T_3_ seeds were harvested. Prior to use in each experiment, T_3_ homozygous seeds were sterilized in 70% (v/v) ethanol with 0.05% (v/v) Triton X-100 for 8 min, washed twice in 100% ethanol for 30 s, and dried at room temperature in a laminar flow hood. Transcript levels of *ScAMT3;3* from 10-d-old homozygous transgenic plants expressing *35S:ScAMT3;3* or *proScAMT3;3:ScAMT3;3* grown on ½ MS media supplemented with 1 mM NH_4_NO_3_ were determined by RT–qPCR with the same primers used for sugarcane, whereas *Arabidopsis Ubiquitin2* (*AtUBQ2*; CCAAGATCCAGGACAAAGAAGGA, TGGAGACGAGCATAACACTTGC, amplicon of 222 bp) was used as a reference gene. Data are expressed as the mean ± SD (*n* = 2, 12 plants per replicate).

### 2.6 Histochemical GUS assay

Seeds of one representative *proScAMT3;3::GUS* homozygous transgenic line were precultured on ½ MS medium for 7 d and then transferred to ½ MS medium containing distinct N concentrations and sources. The N concentrations and sources used were 1 mM NH_4_NO_3_ (+N), 2 mM NH_4_Cl (NH_4_^+^), or no N added (−N). After 14 d, plants were harvested, and the organs were assayed for GUS activity. Samples were transferred to 2-mL microtubes filled with GUS buffer (Jefferson et al., 1987), vacuum-infiltrated, and then incubated in the dark at 37°C for 8 h. The tissue was then cleared in 70% (v/v) ethanol. GUS activity was visualized under a Leica S8AP0 stereomicroscope (Leica Microsystems GmbH, Heidelberg, Germany). For histology, leaf petioles were blocked in basic HistoResin (Leica Biosystems GmbH, Wetzlar, Germany), cross-sectioned in a microtome, mounted with Entelan (Merck KGaA, Darmstadt, Germany), and observed under a Leica DM500 light microscope (Leica Microsystems GmbH).

### 2.7 Phenotypic characterization of transgenic plants

The shoot biomass of *qko* mutant plants transformed with *ScAMT3;3* under the regulation of the sugarcane *ScAMT3;3* native promoter was estimated. Two independent transgenic lines expressing *proScAMT3;3::ScAMT3;3* were precultured on ½ MS medium for 7 d and transferred to ½ MS medium containing various N concentrations and sources for 14 d. The N concentrations and sources used were 2 mM and 10 mM NH_4_Cl or no N added (−N). Plants were harvested after 14 d, and the shoot fresh weight was measured (*n* = 18 biological replicates). The data are expressed as the mean ± SD.

### 2.8 ^15^N–NH_4_^+^ labeling assay

Plants were grown hydroponically for 35 d in a nutrient solution containing 1 mM NH_4_NO_3_, 1 mM KH_2_PO_4_, 1 mM MgSO_4_, 250 µM K_2_SO_4_, 250 µM CaCl_2_, 100 µM Na-Fe-EDTA, 50 µM KCl, 50 µM H_3_BO_3_, 5 µM MnSO_4_, 1 µM ZnSO_4_, 1 µM CuSO_4_, and 1 µM NaMoO_4_. The pH was adjusted to 5.8 with 1 mM KOH (Loqué et al., 2006). For the ^15^N-labeled NH_4_^+^ influx experiment, 35-d-old *qko* mutant plants and two *35S:ScAMT3;3* transgenic lines were transferred to a solution either without N (−N) or with 2 mM NH_4_Cl as the sole source of N for 3 d. The ^15^N–NH_4_^+^ influx in plant roots was conducted after rinsing the roots in 1 mM CaSO_4_ solution for 1 min, followed by incubation for 10 min in a nutrient solution containing 0.1 mM (^15^NH_4_)_2_SO_4_ (60 atm% ^15^N) as the only N source. The roots were then rinsed again in 1 mM CaSO_4_ solution (Loqué et al., 2006). Roots (*n* = 4 biological replicates) were collected, dried, and ground, and 5-10 mg of powder was used for ^15^N determination in an ATLAS MAT CH4 molecular flux mass spectrometer (Laboratório de Isótopos Estáveis, CENA/USP, Piracicaba, SP, Brazil).

For characterization of the NH^4+^ uptake kinetics, 35-d-old *qko* plants and one representative *qko* line expressing *proScAMT3;3::ScAMT3;3* were transferred to a N-deficient nutrient solution for 3 d. After that, roots were treated as described earlier and exposed for 10 min in a nutrient solution containing ^15^N–NH_4_^+^ at concentrations ranging from 0 to 500 µM. ^15^N–NH_4_^+^ influx values were fitted into the Michaelis–Menten equation.

For the ^15^N–NH_4_^+^ remobilization experiment, 35-d-old *qko* mutant plants and two transgenic *qko* lines expressing *proScAMT3;3::ScAMT3;3* were transferred to −N or 2 mM NH_4_Cl solution for 3 d. One non-senescent mature leaf received 10 µL of nutrient solution containing 50 mM (^15^NH_4_)_2_SO_4_ (60 atm% ^15^N) as the sole N source (Fan et al., 2009). The pools of mature and young leaves (*n* = 4 biological replicates) were collected separately, dried, ground, and prepared for ^15^N determination.

For the ^15^N–NH_4_^+^ translocation experiment, 35-d-old *qko* mutant plants and two *qko* transgenic lines expressing *proScAMT3;3::ScAMT3;3* were transferred to a nutrient solution containing 2 mM NH_4_Cl for 3 d. Then, the plants were subjected to ^15^N–NH_4_^+^ influx as described earlier, with the roots placed in a nutrient solution containing 5 mM (^15^NH_4_)_2_SO_4_ (60 atm% ^15^N) as the only N source for 1 h. Subsequently, half of the plant material was harvested, and half was returned to the nutrient solution with 2 mM NH_4_Cl and harvested 24 h after the ^15^N influx. The harvested plant material was divided into roots, mature leaves (leaf 1 to 9), and young leaves (leaf 10 to shoot apex) (*n* = 6 biological replicates) for ^15^N determination.

### 2.9 Statistical analysis

The statistical analyses were performed using R Studio v1.2.5 (http://www.rstudio.com/). The significance between the two groups was calculated using a one-sided Student's t test at p < 0.05. The significance among multiple groups was calculated using one-way ANOVA followed by Tukey's test at p < 0.05.

## 3 Results

### 3.1 The AMT2-type gene *ScAMT3;3* is highly expressed in sugarcane leaves

**Figure 1:**
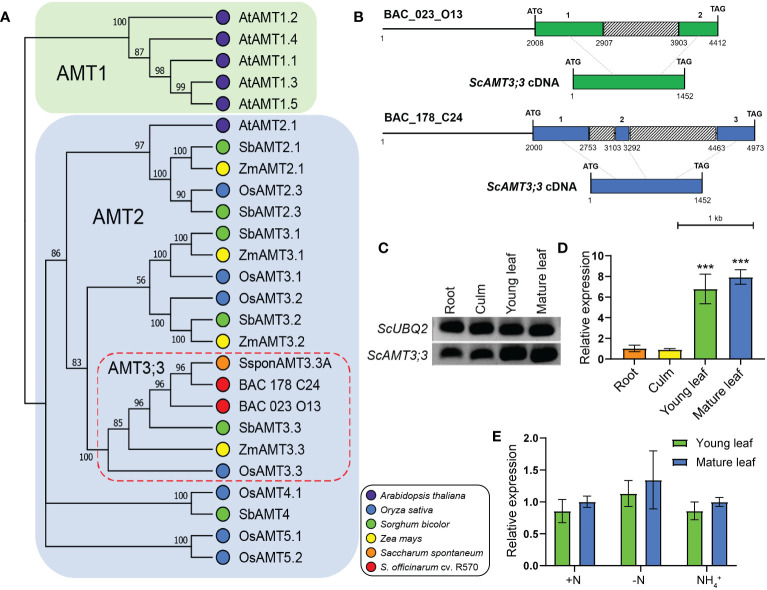
*ScAMT3;3* is an AMT2-type sequence identified in a bacterial artificial chromosome (BAC) library of sugarcane and is highly expressed in leaf tissues. **(A)** Phylogenetic tree of AMTs from *Arabidopsis* and AMT3;3 members from sorghum (*Sorghum bicolor*), maize (*Zea mays*), rice (*Oryza sativa*), sugarcane wild relative *Saccharum spontaneum*, a sugarcane hybrid cultivar (*Saccharum* spp. cv. SP80-3280), and the two putative AMT3;3 sequences found in the BAC library of sugarcane (*Saccharum* spp. cv. R570) are presented. The tree was generated using MEGA X by aligning the complete amino acid sequences of the transporter proteins. The neighbor-joining method was employed with a bootstrap value of 1,000. **(B)** Schematic representation of the structure of the *ScAMT3;3* gene obtained from BAC clones 023_O13 and 178_C24 and their respective cDNAs. The green and blue boxed regions indicate exons, the gray boxes indicate intron regions, and the lines represent the investigated promoter regions. The exon number and the nucleotide coordinates are indicated above and below the boxes, respectively. The scale bar is 1 kb. **(C)** Semiquantitative RT–PCR of *ScAMT3;3* from various tissues of sugarcane cv. SP80-3280 plants grown in nutrient solution containing 1 mM NH_4_NO_3_. **(D)** RT–qPCR analysis of *ScAMT3;3* expression in organs and tissues of sugarcane plants cv. SP80-3280 grown in nutrient solution containing 1 mM NH_4_NO_3_. The mean ± SD (standard deviation; *n* = 4 biological replicates) is presented. Significant differences were observed according to Student’s *t*-test (*p* < 0.001) indicated by ***. **(E)** Transcriptional regulation of *ScAMT3;3* in young and mature leaves according to N status and source. Plants were grown hydroponically for 2 months on 1 mM NH_4_NO_3_ nutrient solution and then transferred to a solution containing 1 mM NH_4_NO_3_ (+N), 2 mM NH_4_Cl (NH_4_^+^), or no N added (−N) for 14d. The mean ± SD (*n* = 4) is presented. Values were standardized for mature leaves collected from +N treatment. No significant differences were observed according to Student’s *t*-test (*p* < 0.05).

Previously, by screening a sugarcane cv. R570 BAC library (Tomkins et al., 1999) for potential AMT2 sequences, 14 clones were retrieved and fully sequenced (Koltun et al., 2022). From these, six BAC clones containing presumed AMT2-type sequences were identified. By aligning their corresponding amino acid sequences with AMT sequences from *Arabidopsis* and grass species, such as sorghum, maize, rice, and *S. spontaneum*, two BAC clones (023_O13 and 178_C24) with coding sequences highly similar to AMT3;3 were identified from grass species (**Figure 1A**; **Supplementary Figure 1**). The AMT coding sequence found in BAC clone 023_O13 was 2,405 bp long (NCBI id OR413321), while that from BAC clone 178_C24 (NCBI id OR413322) was 2,974 bp long (**Figure 1B**). The AMT3;3 coding sequence from BAC clone 023_O13 exhibits two exons, while that from 178_C24 contains three exons, similar to the *S. spontaneum SsAMT3;3* (Wu et al., 2021) and the *Sorghum bicolor SbAMT3;3* (Koegel et al., 2013) (**Figure 1B**; **Supplementary Table 1**). Based on phylogenetic analysis and sequence similarity, we named these two AMT2-type proteins sugarcane AMMONIUM TRANSPORTER 3;3 (ScAMT3;3). The multiple alignments of the two ScAMT3;3 proteins indicated that their identity exceeded 98%, with no notable alteration in the amino acid sequence (**Supplementary Figure 2A**). Additionally, the alignment of the neighboring genomic regions of the BAC clones with *S. spontaneum* and sorghum AMT3;3-type proteins suggested that the ScAMT3;3 sequences found in the BAC library are derived from a duplication event (**Supplementary Figure 2B**).

To gain more information about the regulation of this newly identified AMT, transcriptional profiling of *ScAMT3;3* was performed by semiquantitative RT–PCR and RT–qPCR using roots, culms, young leaves, and mature leaves of sugarcane plants. The transcription pattern of *ScAMT3;3* revealed that this transcript accumulated in all the tissues analyzed, especially in the leaves (**Figure 1C**). The relative expression of *ScAMT3;3* was 6.8- and 7.9-fold higher in young and mature leaves, respectively, than in roots (**Figure 1D**). Because *ScAMT3;3* transcripts were found in larger amounts in the leaves, a detailed analysis of expression was performed in these tissues according to the N status of the plant. Young leaves accumulated slightly fewer *ScAMT3;3* transcripts than mature leaves, and to our surprise, *ScAMT3;3* transcript levels remained stable irrespective of plant N status (**Figure 1E**). Taken together, these results suggest that *ScAMT3;3* is an AMT2-type gene constitutively expressed in aboveground tissues—mainly leaves—and is not regulated by plant N status.

### 3.2 ScAMT3;3 appears to be a transmembrane protein that performs low-affinity NH_4_^+^ transport

**Figure 2:**
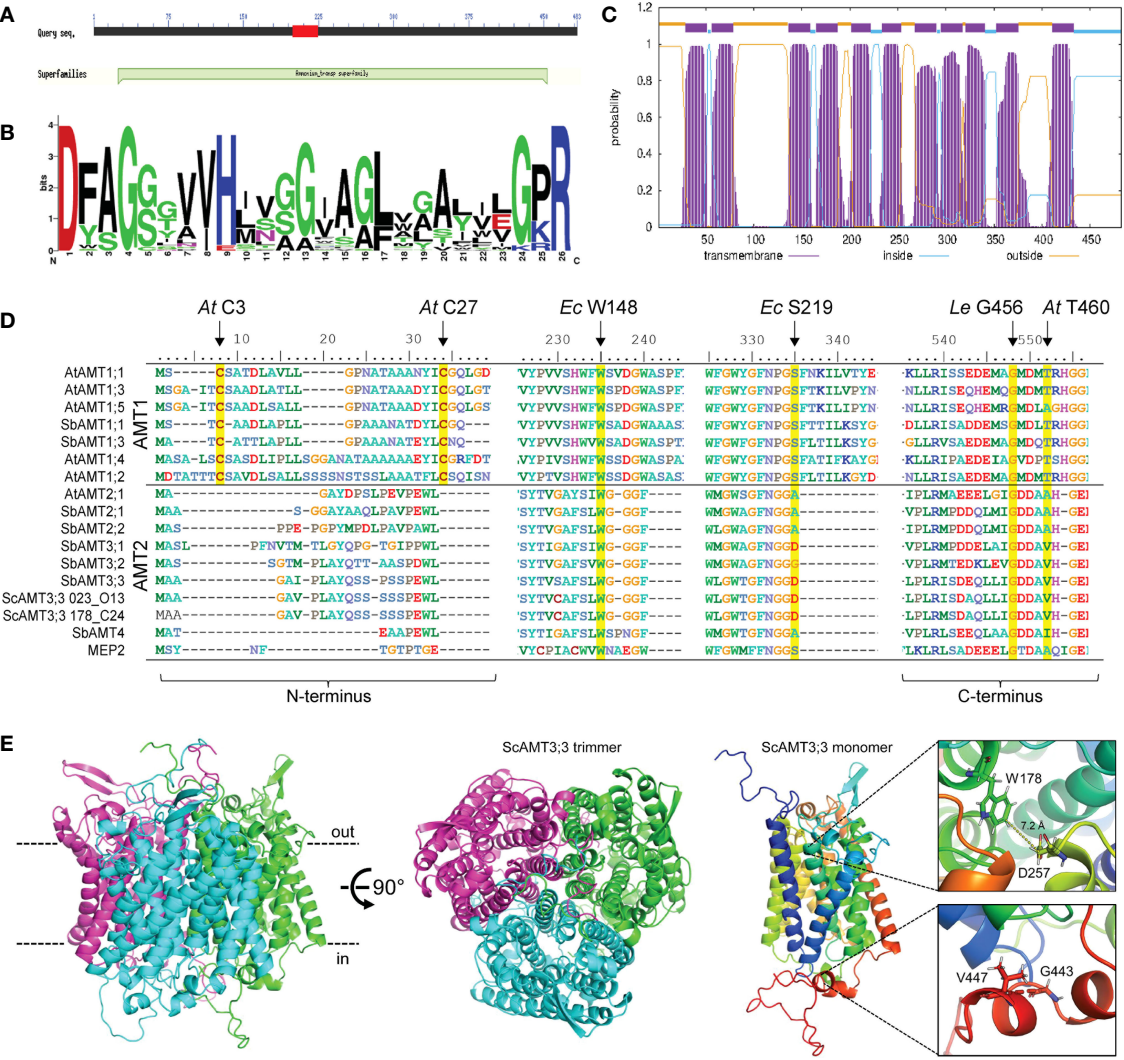
*In silico* analyses of ScAMT3;3 protein sequence. **(A)** Conceptually translated nucleotide sequences identified in BAC clones 023_O13 and 178_C24 from the AMT superfamily. Image was obtained from the CDD server. **(B)** WebLogo for AMT signature motif found in ScAMT3;3 at positions 198–223 based on PS01219 Prosite entry access. **(C)** Transmembrane topology profile prediction of ScAMT3;3 obtained from the clone 023_O13, estimated by TMHMM v.2.0. **(D)** Protein alignment of AMTs from *Arabidopsis* and *Sorghum bicolor*, the two ScAMT3;3 identified in the BAC clones 023_O13 and 178_C24, and MEP2 from *Saccharomyces cerevisiae*, indicating the conservation of critical amino acid residues for function, structure, and regulation of NH_4_^+^ transport. Yellow boxes highlight conserved essential amino acids. *At*, *Arabidopsis thaliana*; *Ec*, *Escherichia coli*; *Le*, *Solanum lycopersicum*. **(E)** Protein structure prediction for ScAMT3;3 from clone 023_O13 obtained by AlphaFold. Inner and outer surfaces of the plasma membrane are depicted as dashed lines in black. The box insets depict a close-up view of the residues W178 (*Ec* W148), D257 (*Ec* S219), V447 (*Le* G456), and G443 (*At* T460).

We conducted an *in silico* analysis of the conceptually translated ScAMT3;3 sequence identified in the BAC clone 023_O13. The ScAMT3;3 identity was validated based on the CDD as an authentic member of the AMT superfamily (**Figure 2A**) since the sequence presented the signature motif for MEP/AMT/Rh, following the pattern D-[FYWS]-[AS]-G-[GSC]-x(2)-[IV]-x(3)-[SAG](2)-x(2)-[SAG]-[LIVMF]-x(3)-[LIVMFYWA](2)-x-[GK]-x-R (Marini et al., 1997; Loqué and von Wirén, 2004), located between residues 198 and 223 (**Figure 2B**). Based on the TMHMM tool, ScAMT3;3 was predicted to contain 11 transmembrane domains, with an apoplastic N-terminus and a cytosolic C-terminus (**Figure 2C**).

We performed multiple alignments of protein sequences to search for highly conserved residues critical for transporter function. Among the conserved amino acids, W148 and S219 from the *E. coli* homolog *EcAmtB* have been shown to be involved in the structure of the NH_4_^+^ binding site (Khademi et al., 2004; Zheng et al., 2004; Andrade et al., 2005). In plant AMT2-type proteins, *Ec* S219 is usually replaced by an Ala or Asp (Pantoja, 2012). Here, we observed that the conserved *Ec* W148 is present in ScAMT3;3 and corresponds to W178 (**Figure 2D**). However, the *Ec* S219 is replaced by D257 in sugarcane, possibly affecting the recruitment of NH_4_^+^ by H-bonding (**Figure 2D**). Another important requirement for AMT function is the formation of a trimer in the plasma membrane (Ludewig et al., 2003; Graff et al., 2011). The presence of the residues Cys3 and Cys27 observed in the N-terminus of the *Arabidopsis* AtAMT1;1 protein is essential for trimerization. Although we did not observe the presence of the conserved Cys in ScAMT3;3 in the N-terminus, which is a common feature in AMT2-type homologs (**Figure 2D**), we were still able to obtain a highly accurate trimer structure (plDDT > 90) when performing a protein structure prediction of ScAMT3;3 using AlphaFold multimer (**Figure 2E**; **Supplementary Figures 3A, B**). Such a protein structure is similar to that observed for MEP2 of yeast, suggesting that trimerization in AMT2-type proteins might be independent of the presence of Cys3 and Cys27. Next, because the presence of residue G456 in *Solanum lycopersicum* LeAMT1;1 and T460 in AtAMT1;1 was shown to be crucial for regulatory function, including posttranslational modifications by phosphorylation (Ludewig et al., 2003; Yuan et al., 2007; Lanquar et al., 2009), we also inspected the C-terminus of ScAMT3;3. Despite the presence of tomato *Le* G456 (G443 in sugarcane) in all the sequences analyzed, *At* T460 was replaced by V447 in ScAMT3;3 (**Figure 2D**). The physiological relevance of this residue substitution for the functionality of ScAMT3;3 remains to be elucidated.

**Figure 3:**
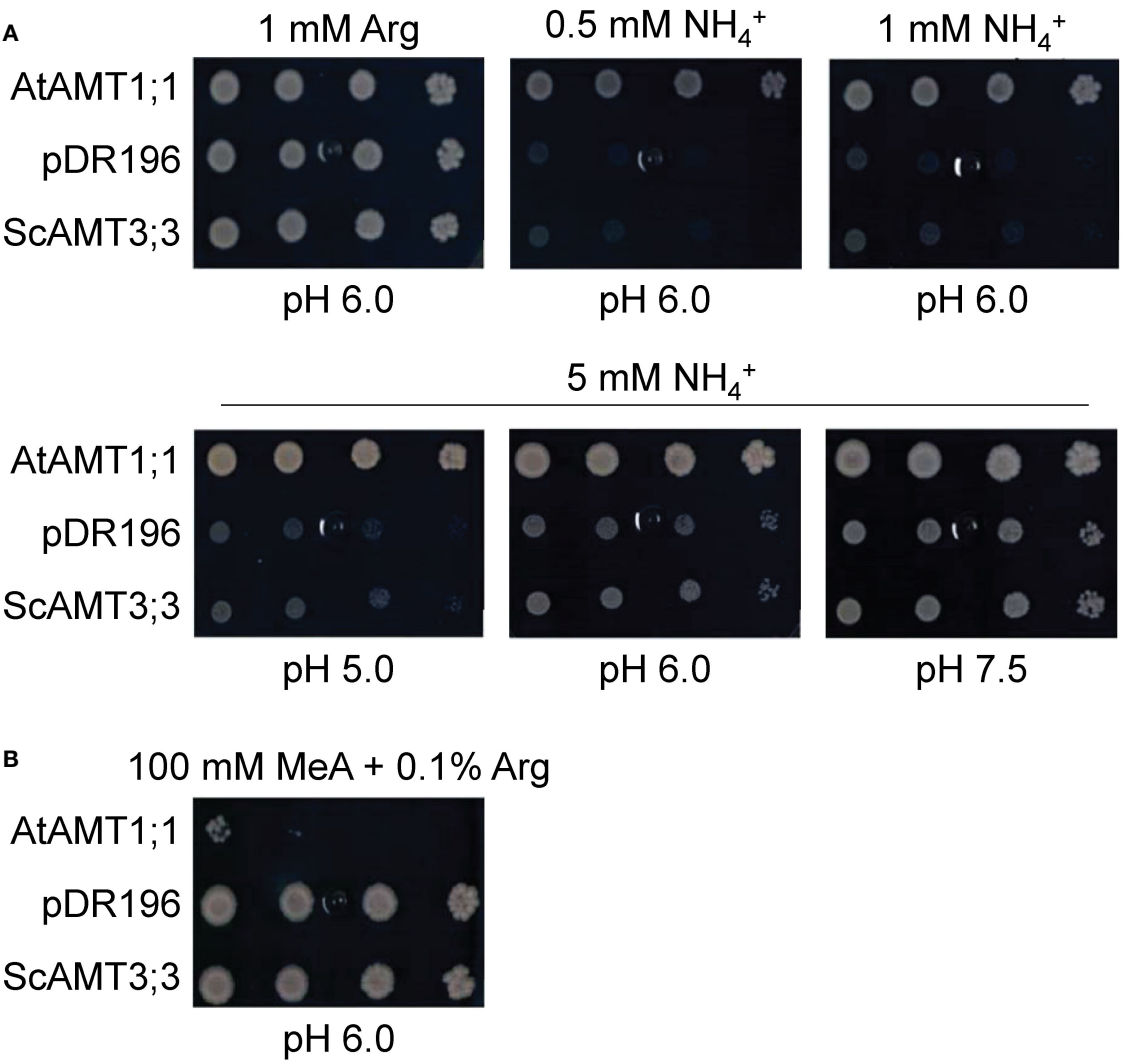
Complementation analysis of ScAMT3;3 protein sequence in yeast defective in NH_4_^+^ transport. **(A)** Functional characterization of ScAMT3;3 in mutant yeast strain 31019b (*MATa ura3 mep1Δ mep2Δ::LEU2 mep3Δ::KanMX2*) (Marini et al., 1997). Growth of AMT-deficient yeast transformed with positive control *AtAMT1;1*, empty pDR196 vector as a negative control, and *ScAMT3;3* on control plates (1 mM arginine; Arg) and selective plates containing 0.5 to 5 mM NH_4_Cl (NH_4_^+^) as sole N sources or 5 mM NH_4_Cl with media pH adjusted to 5.0, 6.0, or 7.5 with 20 mM MES-Tris buffer. Spotted threefold dilutions (10^0^, 10^−1^, 10^−2^, and 10^−3^) of cultures OD_600_ = 1. **(B)** Growth of the same yeast strains on plates containing 100 mM methylammonium (MeA) plus 0.1% Arg.

For functional characterization, we cloned the *ScAMT3;3* sequence from sugarcane cv. SP80-3280 cDNA. This sequence displayed minimal differences with both BAC clone sequences, showing a minimum identity of 99.4%. Additionally, the predicted structures coincided with minimal deviation in the structural alignment (0.05 Å all-atom root mean squared deviation) (**Supplementary Figure 3C**). We then used the sequence to complement the yeast triple *mep* mutant strain 31019b (*mep1*, *mep2*, and *mep3*), which is unable to grow on a medium containing less than 5 mM NH_4_^+^ as the sole N source (Marini et al., 1997). ScAMT3;3 expression complemented the *mep* mutant 31019b, enabling some growth on 5 mM NH_4_^+^ at pH 6.0 (**Figure 3A**; **Supplementary Figure 4**). This result confirmed that ScAMT3;3 is a functional ammonium transporter with a low capacity for NH_4_^+^ transport compared to AtAMT1;1, which belongs to the high-affinity group. Additionally, cells complemented with ScAMT3;3 showed less growth at low pH and better growth at pH 7.5 (**Figure 3A**; **Supplementary Figure 4**). Because the concentration of ammonia (NH_3_) is lower at pH 5.0 than at pH 7.0 and that of NH_4_^+^ remains almost constant, the assay suggests that NH_3_, rather than NH_4_^+^, might be the substrate of ScAMT3;3 transport.

To determine differences in substrate affinities between the AMT proteins, MeA was used as a substrate analog, jointly with 0.1% arginine (Arg). ScAMT3;3-complemented cells were able to grow under 100 mM MeA plus Arg, which corroborated the lower transport capacity for the substrate MeA, whereas AtAMT1;1 presented almost no growth under the same conditions (**Figure 3B**; **Supplementary Figure 4**). These results denote differences in the biochemical properties for NH_4_^+^ recruitment through the membrane by these transporters and that ScAMT3;3 appears to be a functional transporter with affinity to NH_3_.

### 3.3 ScAMT3;3 is functional in plants and is expressed in phloem cells of major veins of leaf tissues

**Figure 4:**
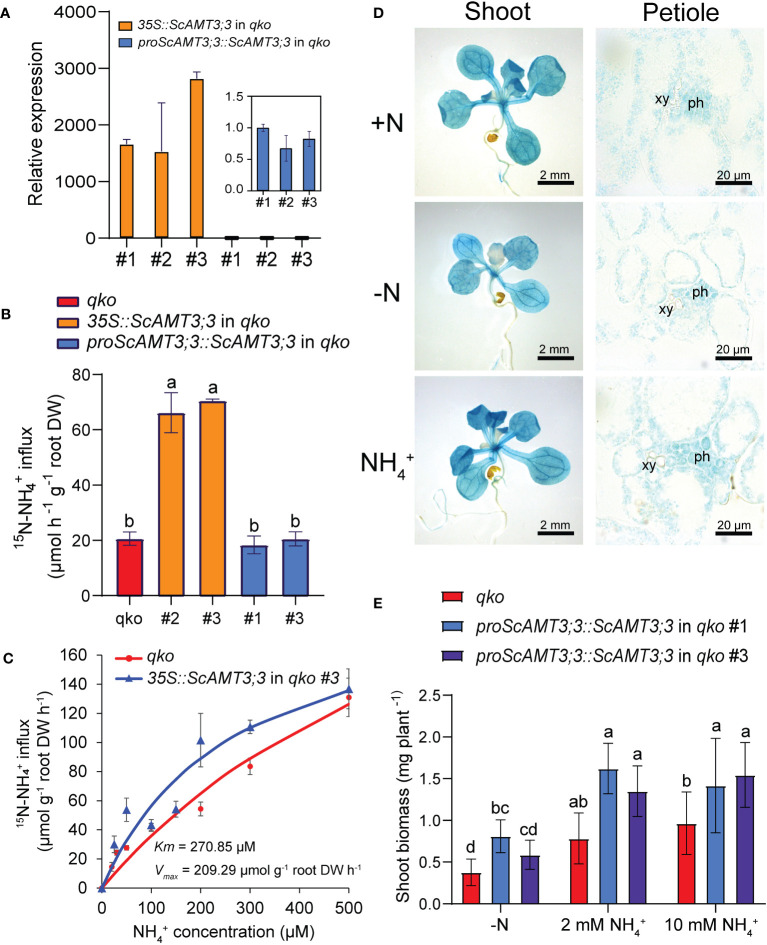
Heterologous expression of ScAMT3;3 in *Arabidopsis*. **(A)** Transcript accumulation of *ScAMT3;3* obtained by RT–qPCR from 10-d-old seedlings of the various *Arabidopsis qko* transgenic lines generated in this study (*35S::ScAMT3;3*, *proScAMT3;3::ScAMT3;3* identified by independent event number #), grown on half-strength Murashige and Skoog (½ MS) media supplemented with 1 mM NH_4_NO_3_. Mean ± SD (standard deviation; *n* = 2; 12 plants per replicate). Values were standardized for *proScAMT3;3::ScAMT3;3* in *qko* #1 plants. **(B)** ^15^N–NH_4_^+^ short-term influx analysis in roots of *qko* and two independent lines of *qko* expressing *35S::ScAMT3;3* or *proScAMT3;3::ScAMT3;3*. Plants were precultivated hydroponically in full-strength nutrient solution supplemented with 1 mM NH_4_NO_3_ for 35 d and transferred to a N-free nutrient solution for 3d. ^15^N-labeled NH_4_^+^ was supplied at 0.1 mM (^15^NH_4_)_2_SO_4_. Mean ± SD (*n* = 4). Different letters indicate significant differences (one-way ANOVA followed by Tukey’s test). **(C)** Concentration-dependent influx of 15N-labeled NH4+ into roots of qko or qko plants expressing 35S::ScAMT3;3. Mean ± SE standard error (n = 6 biological replicates). DW – dry weight. **(D)** Histochemical GUS assay of shoots and cross-sections of leaf petioles of Arabidopsis ‘Col-0’ plants expressing proScAMT3;3::GUS grown on various N sources and availability as indicated below. Plants were precultivated on ½ MS and transferred to plates containing either 1 mM NH4NO3 (+N), 2 mM NH4Cl (NH4+), or N-free (-N) media for up to 14 d. ph – phloem, xy – xylem. **(E)** Shoot biomass of plants expressing *proScAMT3;3::ScAMT3;3* grown on various N sources and availability as indicated below. Plants were precultivated on ½ MS and transferred to media containing N-free (−N) media or 2 mM or 10 mM NH_4_Cl for 14d. Mean ± SD (*n* = 18). Different letters indicate significant differences (one-way ANOVA followed by Tukey’s test).

We then expressed the *ScAMT3;3* coding sequence under the control of both the CaMV35S (*35S::AMT3;3*) or the sugarcane endogenous promoter (*proScAMT3;3::ScAMT3;3*) in the AMT-defective quadruple mutant of *Arabidopsis* (*qko*). The *qko* retains only 10% of the NH_4_^+^ uptake (Yuan et al., 2007). Because *Arabidopsis* plants do not contain any direct *AMT3;3* homolog, we chose one of the *qko* independent transgenic lines expressing *proScAMT3;3::ScAMT3;3* to normalize the relative expression of *ScAMT3;3* transcripts by RT–qPCR. We observed that *35S::ScAMT3;3* plants clearly showed higher levels of transcript accumulation than plants expressing *ScAMT3;3* under its own promoter (**Figure 4A**).

To understand the capacity of ScAMT3;3 to transport NH_4_^+^, roots of N-starved *qko* plants expressing *35S::ScAMT3;3* or *proScAMT3;3::ScAMT3;3* were subjected to short-term influx analysis using ^15^N–NH_4_^+^. *ScAMT3;3*-complemented *qko* plants under the constitutive promoter showed a 3.5-fold increase in uptake of NH_4_^+^ compared to non-transformed *qko* plants (**Figure 4B**). However, when *ScAMT3;3* was regulated by its own endogenous sugarcane promoter, no significant changes in ^15^N–NH_4_^+^ uptake were detected (**Figure 4B**). Since *ScAMT3;3* expression is mainly active in shoots (**Figures 1B, C**) and *qko* plants complemented with *ScAMT3;3* driven by the native promoter showed low transport of NH_4_^+^ (**Figure 4B**), our results suggest that ScAMT3;3 is most likely not critical for NH_4_^+^ uptake in roots.

To further characterize the NH_4_^+^ transport capacity of ScAMT3;3, we estimated the influx rate at increasing concentrations of ^15^N–NH_4_^+^ to obtain the Michaelis–Menten constants (*K_m_* and *V_max_*). Under our experimental conditions, using one independent *qko* line expressing *35S::ScAMT3;3*, we estimated *K_m_* as 270.85 µM and *V_max_* as 209.29 µmol g^−1^ root DW h^−1^ (**Figure 4C**), with an uptake curve similar to that of non-complemented *qko* plants. The data corroborate that ScAMT3;3 participates in the transport of NH_4_^+^ with low affinity, similar to AtAMT2;1 with a *K_m_* value ranging from 36 µM to 3,000 µM (Shelden et al., 2001).

We also conducted tissue-specific expression localization of ScAMT3;3 by fusing 1,877 bp of the 5′-upstream region from the starting ATG of *ScAMT3;3* from the BAC clone 023_O13 to the GUS (*uidA*) reporter gene and transformed *Arabidopsis* ecotype Col-0. To evaluate how the *ScAMT3;3* promoter is regulated by N source and/or supply, we grew one selected transgenic line under various N conditions. Although primary roots exhibited a weak GUS signal, the staining intensity was evidently stronger in the shoots, including cotyledons and leaf tissues (**Figure 4D**). A cross section of the leaf petioles revealed that the promoter activity of *ScAMT3;3* occurs in practically all cell types, but it is more marked in the phloem cell of vascular tissues (**Figure 4D**). When we grew this transgenic line under various N levels and sources, we observed that the *ScAMT3;3* promoter led to slightly lower expression in young leaves under N deficiency, but no visible changes were detected when plants were transferred to NH_4_^+^ as the sole source of N (**Figure 4D**). These results corroborated the higher expression of *ScAMT3;3* observed in leaf tissues of sugarcane (**Figures 1C, D**) and suggested a function of this NH_4_^+^ transporter in leaves.

Since ScAMT3;3 does not appear to be primarily involved in the transport of NH_4_^+^ in roots, we evaluated the contribution of the transporter to shoot biomass accumulation under specific conditions of N source and availability. Irrespective of the N status, the expression of *ScAMT3;3* regulated by its endogenous promoter allowed a predominantly significant increase in shoot biomass accumulation of complemented *qko* plants (**Figure 4E**). Again, these results confirm the importance of ScAMT3;3 in the above-ground tissues. These findings prompted us to hypothesize that this AMT2-type transporter might be involved in internal NH_4_^+^ remobilization to sustain optimal N homeostasis in photosynthetic tissues.

### 3.4 ScAMT3;3 plays a role in NH_4_^+^ transport among source and sink organs upon full N availability

**Figure 5:**
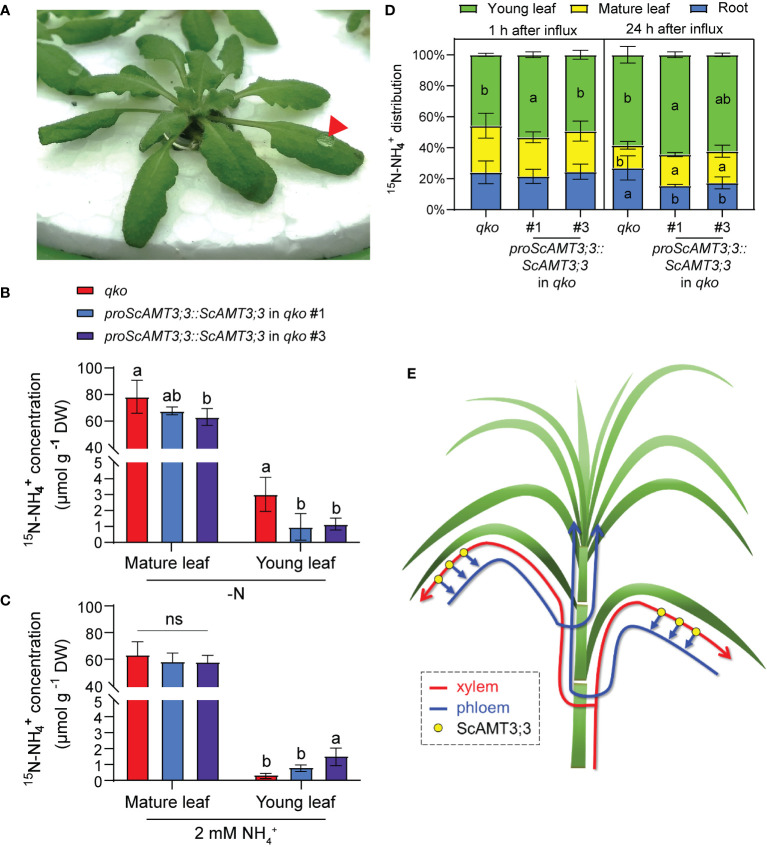
ScAMT3;3 plays an important role in NH_4_^+^ transport among source and sink organs in NH_4_^+^ availability. **(A)** Plant of *Arabidopsis thaliana* grown on hydroponic system to assess ^15^N–NH_4_^+^ remobilization from mature to young leaf. A droplet of 10 μL of 50 mM ^15^(NH_4_)_2_SO_4_ (60 atm%) was applied to a fully expanded mature leaf. The droplet is indicated by a red arrowhead. **(B, C)** Accumulation of ^15^N–NH_4_^+^ in mature and young leaves, representing ^15^N–NH_4_^+^ remobilization in the *qko* mutant, and two independent *qko* complemented lines expressing *proScAMT3;3::ScAMT3;3*. Thirty-five-day-old plants were transferred to N-deficient nutrient solution or containing 2 mM NH_4_Cl for 3d. One fully expanded mature leaf of each plant received a droplet containing ^15^N–NH_4_^+^. After 24 h, a pool of mature and young leaf tissues was collected for ^15^N analysis. Means ± SD (standard deviation; *n* = 4 biological replicates). *p*-Values (one-sided, Student’s *t*-test). **(D)** Distribution of ^15^N–NH_4_^+^ accumulated in roots and mature or young leaves of *qko* and two *qko* independent lines expressing *proScAMT3;3::ScAMT3;3*. Plants were precultivated hydroponically in full-strength nutrient solution (1 mM NH_4_NO_3_) for 35 d and then transferred to a solution containing 2 mM NH_4_Cl as sole N source for 3d. Roots were supplied with 5 mM ^15^(NH_4_)_2_SO_4_ (60 atm%) for 1h. Roots and mature and young leaves were collected after 1 h or 24 h for ^15^N–NH_4_^+^ concentration measurement. Means ± SD (*n* = 6). Different letters indicate statistically significant differences (one-way ANOVA followed by Tukey’s test). **(E)** Proposed model for the role of ScAMT3;3 in translocation of internal NH_4_^+^ in sugarcane. Under NH_4_^+^ supply, this N form is first transported to mature leaves via xylem (red line) and then relocated to young leaves via phloem (blue line). This model was based on the phloem-kickback mode (Yamaji and Ma, 2014).

To verify whether ScAMT3;3 plays a role in the mechanism of N remobilization between mature and young leaf tissues, a droplet of ^15^N–NH_4_^+^ was applied to mature non-senescing leaves of the *qko* mutant and *qko* lines complemented with *proScAMT3;3::ScAMT3;3*, grown hydroponically in N-depleted nutrient solution or containing NH_4_^+^ as the sole source of N (**Figure 5A**). After 24 h, mature and young leaf pools were harvested for ^15^N enrichment analysis. The N-starved *qko* plants tended to show more accumulation of ^15^N–NH_4_^+^ in the mature leaves compared to the complemented lines, possibly due to the inability to relocate this inorganic N form to other organs (**Figure 5B**). Plants from N-deprived complemented lines expressing *ScAMT3;3* accumulated significantly less ^15^N–NH_4_^+^ in the young leaves, suggesting an absence of N flux from source to sink leaves (**Figure 5B**). Conversely, mature leaves from complemented plants expressing *ScAMT3;3*, when grown in nutrient solution containing NH_4_^+^ accumulated 15% to 20% less ^15^N–NH_4_^+^ than those from *qko* plants (**Figure 5C**). In the presence of 2 mM N, the accumulation of ^15^N–NH_4_^+^ was more evident in young leaves from *ScAMT3;*3 complemented *qko* plants, which presented 74% to 86% higher accumulation of ^15^N–NH_4_^+^ compared to the *qko* controls (**Figure 5C**). Taken together, these results suggest that ScAMT3;3 might be involved in the process of NH_4_^+^ remobilization from source to sink organs in the presence of N.

Since ScAMT3;3 function seems to be relevant for NH_4_^+^ transport from source to sink leaf tissues, we evaluated the distribution of ^15^N–NH_4_^+^ in roots, mature leaves, and young leaves from *qko* and *qko* plants complemented by *proScAMT3;3::ScAMT3;3* at 1 h and 24 h after roots were exposed to ^15^N influx at 2 mM NH_4_Cl. After 1 h of influx, mature leaves tended to accumulate ^15^N–NH_4_^+^, but the majority of the N isotope was translocated to young leaves (**Figure 5D**). The accumulation of ^15^N–NH_4_^+^ in the young leaves of complemented *proScAMT3;3::ScAMT3;3* plants was 29% to 72% higher than that in the *qko* mutant, suggesting that the activity of ScAMT3;3 may assist in the translocation of NH_4_^+^ to these tissues. After 24 h of influx, the accumulation of ^15^N–NH_4_^+^ tended to be higher in both mature and young leaves of ScAMT3;3-complemented plants than in those of the *qko* genotype (**Figure 5D**). The higher accumulation of ^15^N–NH_4_^+^ observed in plants after 24 h of influx may be due to either the redistribution of NH_4_^+^ via phloem between source and sink tissues or the assimilation of ^15^N–NH_4_^+^ into amino acids by isoforms of glutamine synthetase in phloem cells.

## 4 Discussion

Sugarcane is a traditional global crop that is recognized as an efficient producer of biomass on an industrial scale. In addition to its traditional products sugar and bioethanol, novel uses of sugarcane byproducts, such as the potential development of green hydrogen from bagasse (Formann et al., 2020; Chatterjee and Mohan, 2021), warrant the anticipated increase in demand for sugarcane cultivation, possibly in marginal soils. However, the species exhibits a limited N uptake capacity (Lima et al., 2022). The crop will require more N fertilization to achieve competitive yields, which could give rise to considerable environmental challenges typical of N fertilization (Martìnez-Dalmau et al., 2021). AMTs are important components of the N uptake and transport systems and are crucial for sustaining effective growth and yield (Bloom, 2015; Fan et al., 2016; Fan et al., 2017; Perchlik and Tegeder, 2017). Consequently, elucidating the role of specific AMTs is critical for devising strategies to enhance crop NUE and the overall improvement in sustainable sugarcane cultivation practices.

To date, extensive research has been carried out to explore the roles of AMT-type transporters in NH_4_^+^ nutrition in model plants and a few crop species (Marini et al., 1994; Gazzarrini et al., 1999; von Wirén et al., 2000; Suenaga et al., 2003; D’Apuzzo et al., 2004; Couturier et al., 2007; Gu et al., 2013; Koegel et al., 2013). In sugarcane, our group previously documented the involvement of ScAMT2;1 in NH_4_^+^ translocation from roots to shoots (Koltun et al., 2022). In the present work, we identified and functionally characterized a novel AMT2-type gene from the sugarcane cultivar R570, namely, *ScAMT3;3*, presumably involved in the transport of N from source to sink tissues.

AMT2 proteins appear to have arisen from a horizontal gene transfer event, likely from an *Archaea* MEP-type subfamily (McDonald et al., 2012) and later expanded in land plant genomes (Von Wittgenstein et al., 2014). Evolutionary analyses suggested that the *Saccharum AMT* gene family may have expanded via whole-genome duplication events, which includes AMT2-type genes (Wu et al., 2021). Indeed, by screening clones from a sugarcane genome BAC library (Tomkins et al., 1999), we were able to identify six clones positive for *AMT2*-type genes, of which two clones contained *AMT3;3* homologs to other grass species, and we named these two *AMT3;3* sequences *ScAMT3;3*. Our *in silico* analysis of the conceptually translated ScAMT3;3 identified the highly conserved domain and residues typical of AMTs and pointed to a typical transmembrane topology with the ability to form a trimer structure in the plasma membrane according to a prediction by AlphaFold.

Furthermore, NH_4_^+^ transport capacity analysis using mutant yeast complementation demonstrated that sugarcane ScAMT3;3 proteins are functional in NH_4_^+^ transport, but not with high affinity. In fact, our estimation of *K_m_* (270.85 µM) and *V_max_* (209.29 µmol g^−1^ root DW h^−1^) suggested that ScAMT3;3 participates in the transport of NH_4_^+^ with low affinity (Shelden et al., 2001). By ectopic expression of *ScAMT3;3* in *Arabidopsis qko* plants, we restored the transport capacity of ^15^N–NH_4_^+^ in roots and allowed plant growth under specific N availability conditions, confirming the function of *ScAMT3;3* in NH_4_^+^ transport *in planta*. In addition, our results showed a novel physiological feature of AMT2-type transporters, which appears to substantially contribute to the leaf-to-sink translocation of NH_4_^+^.

The allocation and redistribution of N between source and sink tissues, in conjunction with the regulation of growth in response to varying N supply levels and developmental stages, are essential components of plant physiology (Malagoli et al., 2005; Diaz et al., 2008; Santiago and Tegeder, 2017; Tegeder and Masclaux-Daubresse, 2018). This dynamic process primarily relies on the phloem for the transport of N from source leaves to sink tissues (Van Bel, 1990). Notably, model plants like *Arabidopsis* and many crops, including sugarcane, predominantly employ apoplastic phloem loading mechanisms (Rennie and Turgeon, 2009). Within this complex process, the release of organic and/or inorganic N compounds from parenchyma or bundle sheath cells into the leaf apoplast occurs passively, followed by active transport mediated by plasma membrane proteins to import N into the phloem (Okumoto and Pilot, 2011; Tegeder et al., 2012; Hsu and Tsay, 2013). N allocation and redistribution play an important role in plant growth, and the transport of N from source to sink tissues occurs mainly by the phloem (Van Bel, 1990; Malagoli et al., 2005; Diaz et al., 2008; Masclaux-Daubresse et al., 2010; Santiago and Tegeder, 2017). Additionally, amino acid catabolism and photorespiratory recycling alter NH_4_^+^ concentrations (Brugière et al., 1999; Masclaux et al., 2000; Diaz et al., 2008), and the degradation of chloroplasts and glutamate dehydrogenase can contribute to the accumulation of NH_4_^+^. These interrelated processes collectively control the efficient use and distribution of N in plants, ultimately impacting their growth and development.

To maintain optimal NH_4_^+^ homeostasis, plants employ a series of regulatory mechanisms, such as the regulation of transcription levels of AMTs. Here, we analyzed tissue-specific expression levels of *ScAMT3;3* in sugarcane under N-sufficient or N-deficient conditions. Our gene expression analysis revealed that *ScAMT3;3* is predominantly expressed in the leaves of sugarcane rather than in the roots or culms. Furthermore, no significant variation in *ScAMT3;3* expression was detected in sugarcane leaves according to plant N status. Previously, *SsAMT2;1* and *SsAMT3;2* were shown to be highly expressed in leaves of sugarcane hybrid cultivars YT55 and YT00–236 (Wu et al., 2021). Additionally, *OsAMT2;1* and *ZmAMT3;2* were found to be mainly expressed in old leaves, suggesting their possible role in the translocation of NH_4_^+^ from old to young leaves (Su-Mei et al., 2012; Dechorgnat et al., 2019). Using a heterologously expressed GUS reporter gene in *Arabidopsis* driven by a sugarcane *AMT3;3* promoter, we observed signaling of *ScAMT3;3* promoter activity in the leaf tissue, particularly in phloem vascular cells. Altogether, we hypothesized that ScAMT3;3 may be involved in long-distance NH_4_^+^ translocation from source leaves to sinks.

Using the mature leaf ^15^N–NH_4_^+^ feeding approach to assess NH_4_^+^ source-to-sink distribution in transgenic *qko* mutant plants expressing *ScAMT3;3* driven by its endogenous sugarcane promoter showed that, irrespective of the N status of the plant, NH_4_^+^-fed *qko* plants harboring *proScAMT3;3::ScAMT3;3* displayed more NH_4_^+^-derived ^15^N allocation to young leaves than *qko* plants. During vegetative growth, both roots and developing leaves function as strong N sinks. By modulating the N status of *qko* plants expressing *proScAMT3;3::ScAMT3;3*, we observed an increased allocation of NH_4_^+^-derived ^15^N to developing young leaves when plants were cultivated under N-sufficient conditions. Conversely, under N-deficient growth conditions, ^15^N–NH_4_^+^ allocation to young leaves was reduced compared to that in *qko* control plants, suggesting that ^15^N might be allocated to roots under a limited N supply (**Supplementary Figure 5**). In an independent experiment to address leaf-to-sink allocation through ^15^N-long-term labeling of roots in plants, which allows the determination of N fluxes, *qko* plants expressing *proScAMT3;3::ScAMT3;3* contained significantly more NH_4_^+^-derived ^15^N allocation to young leaves than *qko* plants after 24 h. Both experimental settings demonstrated that ScAMT3;3-dependent ^15^N–NH_4_^+^ phloem loading might be a major control point in source-to-sink allocation. In agreement with the phloem-kickback model (Yamaji and Ma, 2014), we propose that in sugarcane, NH_4_^+^ is first distributed to the expanded mature leaf and then redistributed to the developing young tissues via phloem transport (**Figure 5E**).

In conclusion, this study revealed the potential role of ScAMT3;3 in NH_4_^+^ transport and long-distance NH_4_^+^ translocation in sugarcane. As sugarcane assumes a pivotal role in biomass production and alternative energy generation, understanding the mechanisms underlying its N utilization becomes imperative for sustainable cultivation. The identification and functional characterization of ScAMT3;3 provide valuable insights into potential strategies for optimizing NUE in sugarcane cultivars. Additionally, this study contributes to the broader understanding of AMT2-type transporters and their evolutionary significance in plants. These findings collectively advance our knowledge of N transport in plants and its implications for agricultural sustainability and bioenergy production.

## Data availability statement

Cited sequence data can be found at the EMBL/GenBank data libraries under accession number(s): AtAMT1;1 NP_193087.1, AtAMT1;2 AEE34288.1, AtAMT1;3 AEE76886.1, AtAMT1;4 Q9SVT8.1, AtAMT1;5 Q9LK16.1, AtAMT2;1 ABF57277.1, OsAMT2;3 NP_915337.1, OsAMT3;1 BAC65232.1, OsAMT3;2 AAO41130, OsAMT3;3 Q69T29, OsAMT4;1 Q10CV4, OsAMT5;1 Os12g01420.1, OsAMT5;2 Os11g01410.1, SbAMT2;1 Sb09g023030.1, SbAMT2;2 Sb03g038840.1, SbAMT3;1 Sb03g041140.1, SbAMT3;2 Sb01g001970.1, SbAMT3;3 Sb04g022390.1, SbAMT4 Sb01g008060.1, SsponAMT3;3 04G0010470_2B, ZmAMT2;1 GRMZM2G080045, ZmAMT3;1 GRMZM2G335218, ZmAMT3;2 GRMZM2G338809, ZmAMT3;3 GRMZM2G043193, and ScAMT3;3 OR413321 and OR413322.

## Author contributions

RM: Investigation, Methodology, Writing – original draft. AK: Investigation, Methodology, Writing – review & editing. MV: Methodology. BF: Methodology. NS: Investigation, Formal Analysis, Methodology, Writing – review & editing. AC: Methodology, Writing – review & editing. JL: Methodology, Conceptualization, Funding acquisition, Investigation, Resources, Supervision, Writing – review & editing. AF: Funding acquisition, Resources, Supervision, Writing – review & editing.
