# Supplementary material for: Identification and functional characterization of the sugarcane (Saccharum spp.) AMT2-type ammonium transporter ScAMT3;3 revealed a presumed role in shoot ammonium remobilization
Source: Front Plant Sci. 2023 Nov 30;14:1299025. doi: 10.3389/fpls.2023.1299025 (PMC10720369; doi:10.3389/fpls.2023.1299025)
Supplement: Supplementary file 1 [file DataSheet_1.pdf]

Identification and functional characterization of the sugarcane (*Saccharum* spp.) AMT2-type ammonium transporter ScAMT3;3 revealed a presumed role in shoot ammonium remobilization

Rodolfo A. Maniero[1,4†], Alessandra Koltun[1†], Marielle Vitti[1], Bruna G. Factor[1], Nathalia de Setta[2], Amanda S. Câmara[4], Joni E. Lima[3,*], Antonio Figueira[1,*]

[1] Centro de Energia Nuclear na Agricultura, Universidade de São Paulo, Piracicaba, SP, Brazil

[2] Centro de Ciências Naturais e Humanas, Universidade Federal do ABC, São Bernardo do Campo, SP, Brazil

[3] Instituto de Ciências Biológicas, Universidade Federal de Minas Gerais, Belo Horizonte, MG, Brazil

[4] Leibniz Institute of Plant Genetics and Crop Plant Research (IPK), Gatersleben, Seeland, Germany

[†] These authors have contributed equally to this work

[*] Corresponding author: limajoni@googlemail.com; figueira@cena.usp.br

Email addresses: rodolfo.maniero@alumni.usp.br; koltun@alumni.usp.br; marielle.vitti@gmail.com; bruna.factor@usp.br; nathalia.setta@ufabc.edu.br; camara@ipk-gatersleben.de; limajoni@googlemail.com; figueira@cena.usp.br

Supplementary Figures

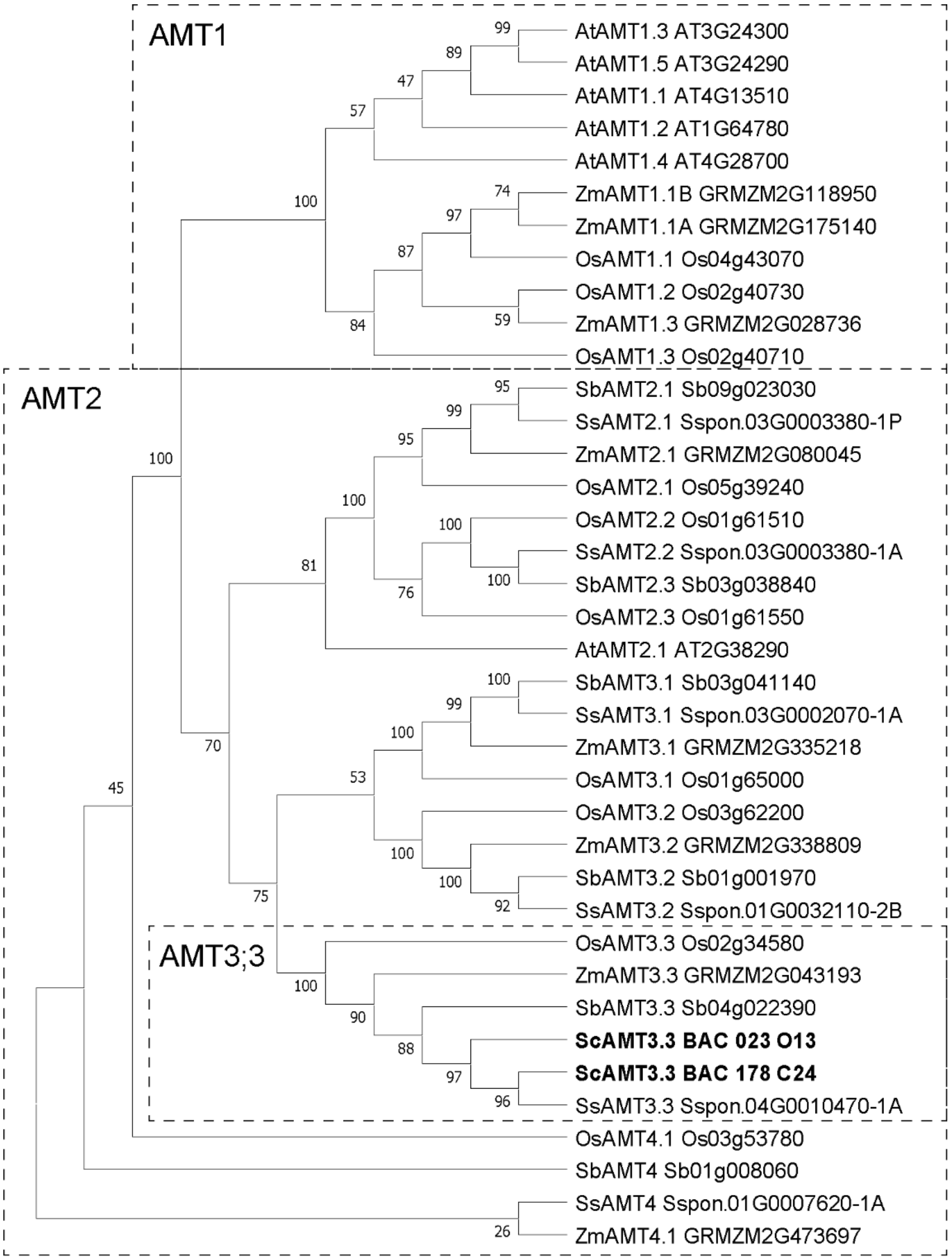

Supplementary Figure S1. Expanded phylogenetic tree of AMTs from several plant species, including *A. thaliana* (At), *Oryza sativa* (Os), *Sorghum bicolor* (Sb), *Zea mays* (Zm), *S. spontaneum* (Ss), and the two clones BAC from *S. officinarum* cultivar 'R750' (Sc). Protein sequences were aligned using MAFFT 7 and the phylogenetic tree was built using the neighbor-joining method by MEGA X, with 1000 bootstrap replicates. Each protein name is followed by accession numbers/locus ID; BAC clone 023_O13 accession number is OR413321 and 178_C24 is OR413322. Dashed boxes highlight the subfamilies AMT1 and AMT2, and the AMT3;3 clade.

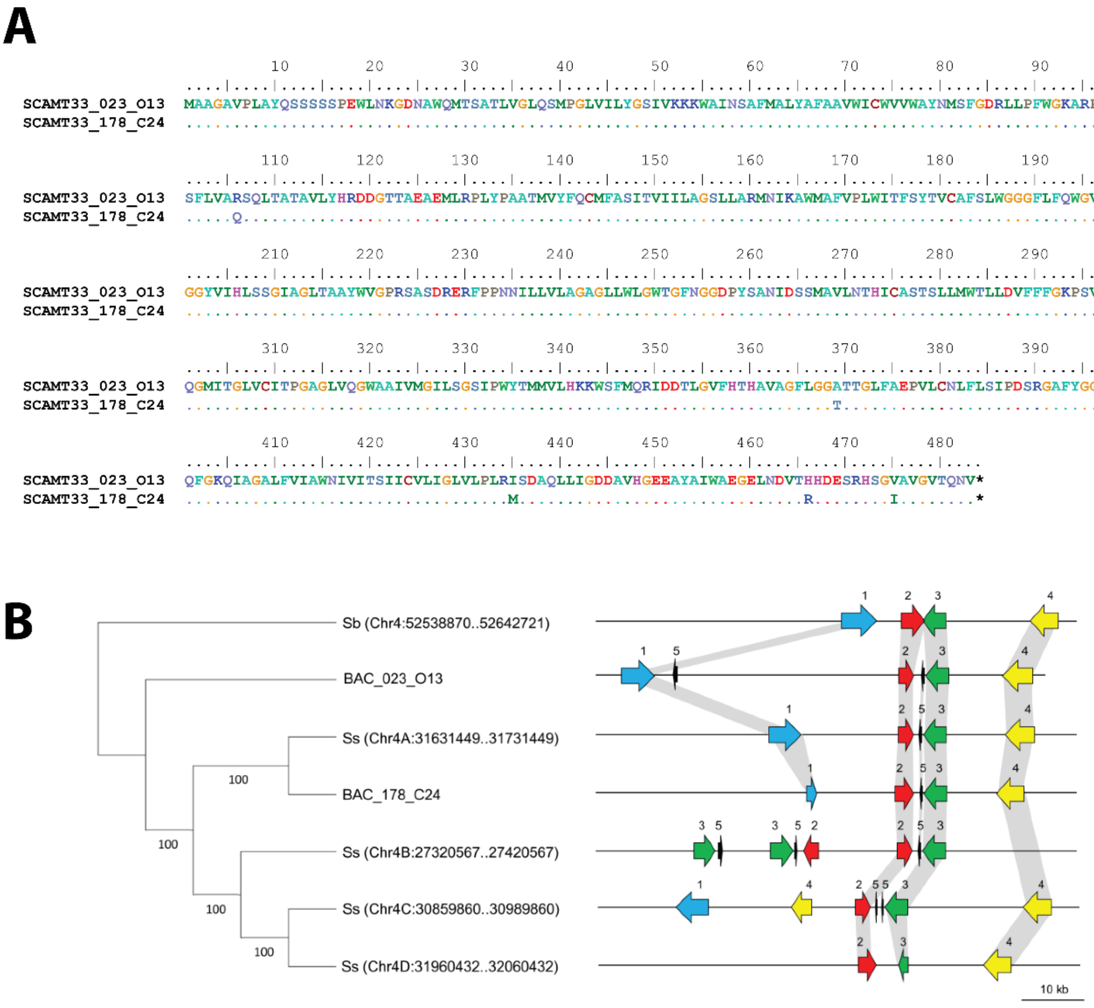

Supplementary Figure S2. Alignment of putatively translated sequences of ScAMT3;3 identified in the BAC library and the physical map of genomic sequences. (**A**) ScAMT3;3 conceptually translated sequences from BAC clone 023_O13 aligned with ScAMT3;3 from BAC clone 178_C24, indicating 98.96% of identity and 99.38% of similarity. (**B**) Phylogenetic tree generated using MEGA X by aligning the proximal genomic sequences of the transporter proteins including *AMT3;3* from sugarcane (*Saccharum* spp. cv. R570) BAC clones (023_O13 and 178_C24); *S. bicolor* (chromosome 4, Sb04g022390); and *S. spontaneum* (chromosome 4, Sspon.04G0010470-1A, Sspon.04G0010470-2B, Sspon.04G0010470-3C). The neighbor-joining method was employed with a bootstrap value of 1,000. Gene models identified in the genomic sequences: 1 (blue arrow) - *EXOSOME COMPLEX COMPONENT RRP45A-LIKE*; 2 (red arrow) - *AMMONIUM TRANSPORTER 3 MEMBER 3*; 3 (green arrow) - *PROTEIN FAR1-RELATED SEQUENCE 5-LIKE*; 4 (yellow arrow) - *SERINE/THREONINE-PROTEIN KINASE SAPK7*; 5 - (black arrow) *CYTOCHROME P450*.

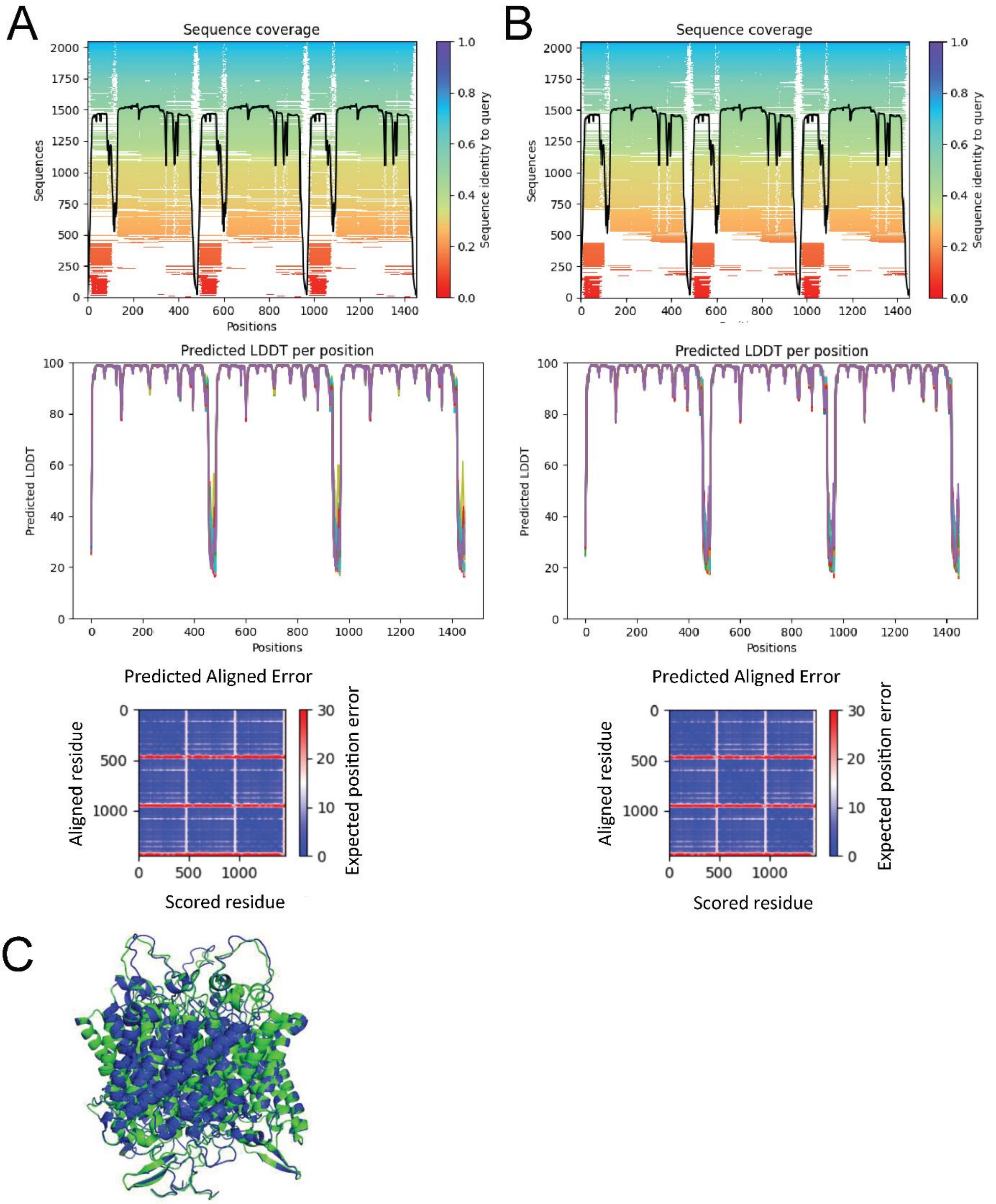

Supplementary Figure S3. Confidence scores for the predicted structures of ScAMT3;3 trimer by AlphaFold multimer. Predictions were made separately for BAC clones (**A**) 013_O23 and (**B**) 178_C24. Residue positions are relative to the homotrimer. Top figures show the sequence coverage, where each line represents one sequence in the Multiple Sequence Alignment used. Graphics in the middle indicate the predicted Local Distance Difference Test for each model, 25 in total indicated by different colors. Bottom figures show the Predicted Aligned Error indicating accurate inter-monomer positioning. (**C**) Structural alignment of the best predicted structures for both clones, one in green and the other in blue.

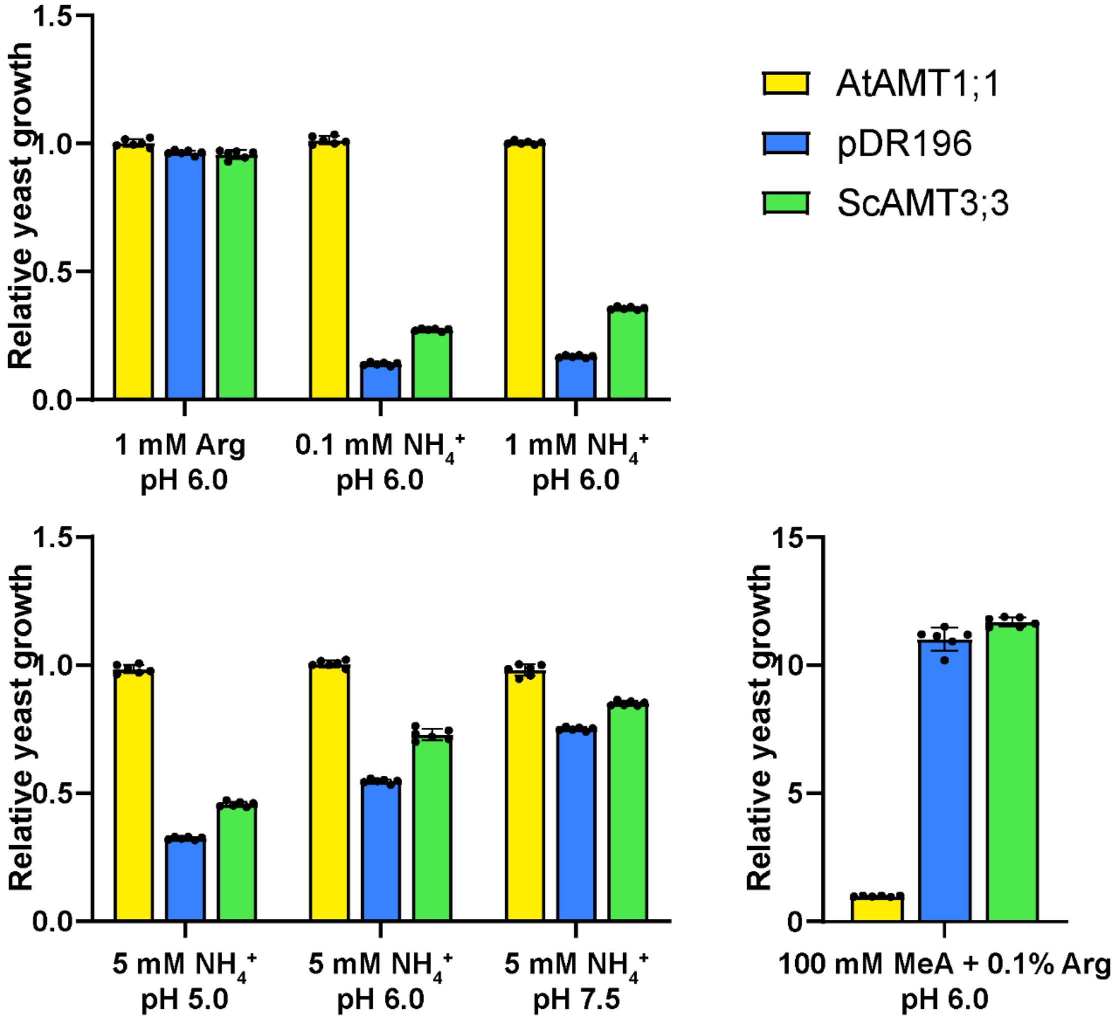

Supplementary Figure S4. Quantitative evaluation of yeast growth from spotting assay shown in Fig. 3. Growth of AMT-deficient yeast transformed with the empty pDR196 vector as a negative control or *ScAMT3;3* in relation to yeast transformed with *AtAMT1;1* as positive control. Data was obtained by employing an imaging-based quantification method (Petropavlovskiy et al., 2020). The dilution 10^{-1} was chosen for the quantification. Means ± SD (*n* = 6; standard deviation). Arg, arginine; NH_4^+, NH_4Cl; MeA, methylammonium. Media pH was adjusted to 5.0, 6.0, or 7.5 with 20 mM MES-Tris buffer.

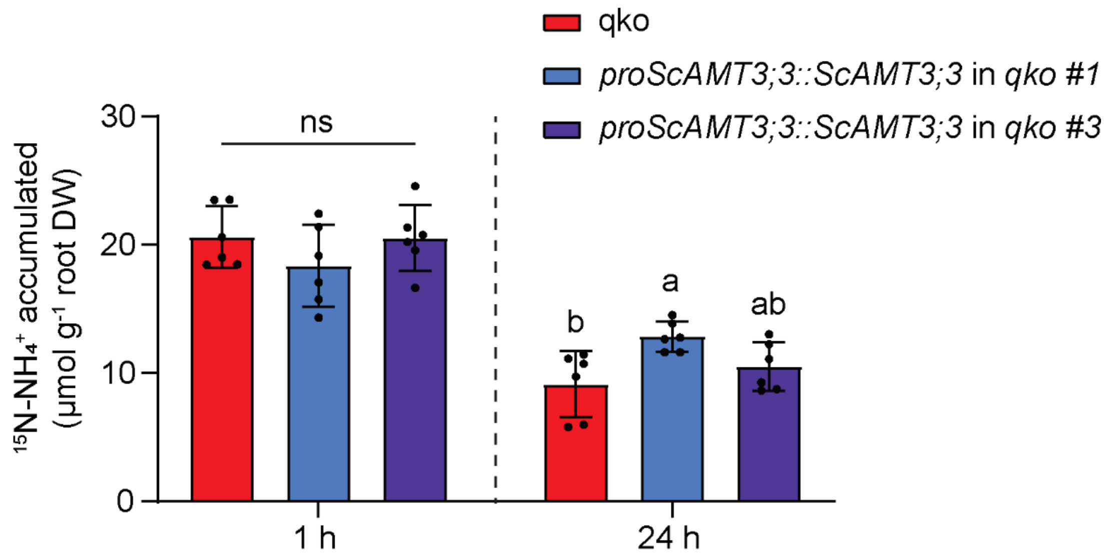

Supplementary Figure S5. Accumulation of ^{15}N-NH_4^+ in roots of *qko* mutant, and two independent *qko* complemented lines expressing *proScAMT3;3::ScAMT3;3*. Thirty-five-d old plants were transferred to N-deficient nutrient solution for 3 d. Next, roots were supplied with 5 mM $^{15}(NH_4)_2SO_4$ (60 atm%) for 1 h. Roots were collected after 1 h or 24 h for ^{15}N-NH_4^+ level measurement. Means ± SD (n = 6). Different letters indicate statistically significant differences (one-way ANOVA followed by Tukey's test).

Supplementary Table S1. Features of *AMT3;3* identified in *S. spontaneum* and BAC clone from *S. officinarum* (cultivar 'R570'). Conceptually translated protein molecular weight and isoelectric point of protein were estimated with Expasy (https://web.expasy.org/compute_pi/). Number of transmembrane domains was predicted with TMHMM Server v.2.0 (http://www.cbs.dtu.dk/services/TMHMM/). Similarity was calculated with Sequence Manipulation Suite (https://www.bioinformatics.org/sms2/ident_sim.html). Subcellular localization was predicted using WoLF PSORT (https://www.genscript.com/wolf-psort.html). Plas, plastid; Vacu, vacuole.

**Gene name**	*SsAMT3.3*	*ScAMT3;3*	*ScAMT3;3*
**Gene locus ID**	Sspon. 04G0010470-1A	OR413321	OR413322
**CDS length (bp)**	1557	1452	1452
**Number of introns**	2	3	2
**Number of amino acids**	519	483	483
**Molecular weight (kDa)**	55.49	52.02	52.07
**Isoelectric point (pI)**	7.04	6.04	5.97
**Number of transmembrane domains**	11	11	11
**Subcellular localization**	Plas/Vacu	Plas/Vacu	Plas/Vacu
**Sorghum ortholog ID**	Sobic. 004G173200	Sobic. 004G173200	Sobic. 004G173200
**Similarity (%)**	96.2	97.3	97.5
